# Two Rationally Identified Novel Glitazones Reversed the Behavioral Dysfunctions and Exhibited Neuroprotection Through Ameliorating Brain Cytokines and Oxy-Radicals in ICV-LPS Neuroinflammatory Rat Model

**DOI:** 10.3389/fnins.2020.530148

**Published:** 2020-09-25

**Authors:** Antony Justin, Premkumar Ashwini, Jincy A. Jose, Victoria Jeyarani, S. P. Dhanabal, Chennu Manisha, Subhankar P. Mandal, Guru Bhavimani, P. Prabitha, S. Yuvaraj, B. R. Prashantha Kumar

**Affiliations:** ^1^Department of Pharmacology, JSS Academy of Higher Education & Research, JSS College of Pharmacy, Ooty, India; ^2^Department of Pharmaceutical Chemistry, JSS Academy of Higher Education & Research, JSS College of Pharmacy, Mysuru, India

**Keywords:** neuroinflammation, PPAR-γ, glitazones, PGC-1α, cytokines, anti-oxidant

## Abstract

The present study has planned to evaluate the neuroprotective activity of two novel glitazones in a neuroinflammatory rat model. Two novel glitazones were selected from an in-house virtual library of glitazones based on their docking scores against peroxisome proliferator-activated receptor-gamma (PPAR-γ) protein and other parameters studied in *in silico* computational studies. Initially, an acute oral toxicity study was carried out for glitazones in rats to assess the toxicity profile and to determine the therapeutic range for neuroprotective evaluation. Prior to induction of neuroinflammation, the treatments with glitazones (G1 and G2) and standard pioglitazone were made for four consecutive days to respective groups. On the fifth day, the neuroinflammation was induced by intracerebroventricular (ICV) administration of lipopolysaccharides (LPS) (2 μg/μl) using stereotaxic apparatus. After 7 days, the rats were subjected to behavioral assessment followed by neurobiochemical evaluation and histopathological studies. The pre-treatment with glitazones at two dose levels (15 and 30 mg/kg) has significantly reversed behavioral dysfunctions. Glitazones have shown significant reduction in the levels of LPO, NO, TNF-α, and IL-1β and also increased the levels of antioxidant enzymes such as SOD, CAT, and GSH in the brain of LPS-administered rats. The neuroprotection exhibited by two novel glitazones is comparable with standard pioglitazone. The PPAR-γ-dependent amelioration of cytokines and oxy-radicals released by novel glitazones during neuroinflammatory conditions may be attributed to the reversal of behavioral dysfunctions through preventing the degeneration of neurons in major regions of the brain.

## Introduction

Neuroinflammation contributes to many neurodegenerative disorders such as Alzheimer’s disease (AD), Parkinson’s disease (PD), amyotrophic lateral disorders, and multiple sclerosis (MS) (Prathab Balaji et al., 2015). Inflammation in nerve tissues is mainly mediated through activation of microglial cells, which are the resident brain macrophages and regulate the release of inflammatory cytokines and oxy-radicals. The initial protective response of the brain is neuroinflammation, but the excessive inflammatory responses result in neurodegeneration (Gonzalez et al., 2014; Zhang and Jiang, 2015). Factors like aging, trauma, dementia, stroke, depression, hypertension, diabetes, tumors, toxins, and infections are the major risk factors that contribute to neuroinflammation.

Acute neuroinflammation may disappear within a short period of time or advance to chronic inflammation. Chronic inflammation in the brain tissue contributes to several neurological disease conditions based on the site of inflammation (Kempuraj et al., 2016). Therefore, neuroinflammation plays an imperative role in several neurodegenerative disorders and may have a high therapeutic impact especially in the clinical treatment. Studies have revealed that agonist activity at peroxisome proliferator-activated receptor-gamma (PPAR-γ) has beneficial effect in neurodegenerative diseases through upregulating PGC-1α expression and attenuating inflammation, oxidative stress, and mitochondrial dysfunction (Rona-Voros and Weydt, 2010).

Peroxisome proliferator-activated receptor-gamma is a ligand-activated transcriptional factor of nuclear receptor super family. It controls the expression or activity of many genes involved in a variety of cell signaling pathways, including glucose homeostasis, regulation of insulin sensitivity, fatty acid metabolism, immune responses, and inflammation. PPAR-γ is expressed in various cell types, such as immune cells, adipose tissues, and brain cells including microglia and astrocytes, which contribute to anti-inflammatory response (Corona and Duchen, 2016). The use of PPAR-γ agonist was found to be advantageous in many neurodegenerative disorders. It has been reported that pioglitazone belongs to the glitazone family and PPAR-γ agonist has significantly attenuated the lipopolysaccharide (LPS)-induced production of cytokines and oxy-radicals from microglial cells (Chuang et al., 2016). Also, pioglitazone significantly inhibited the LPS-induced expression of induced nitric oxide synthase (i-NOS) and nitric oxide (NO) production (Xing et al., 2008).

**FIGURE 1:**
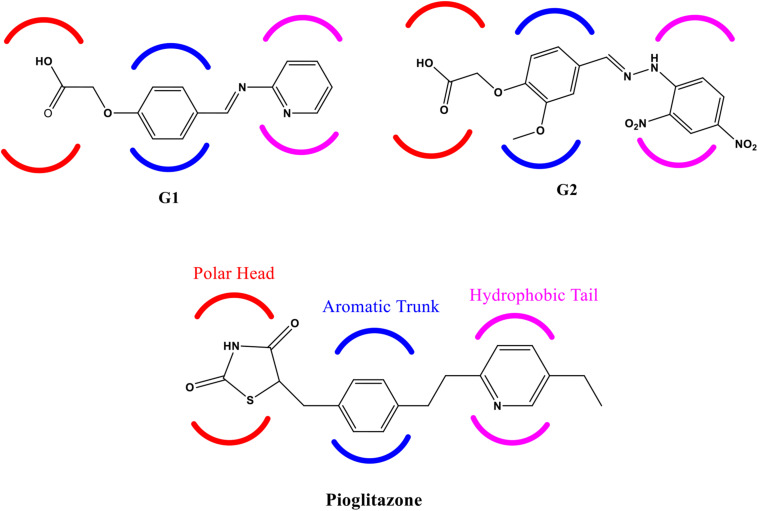
Structure of Glitazone 1 (G1), Glitazone 2 (G2), and standard pioglitazone with conserved regions for binding to PPAR-γ receptor protein.

Another experimental finding has shown that PPAR-γ agonist potentially down-regulated the three pro-inflammatory cytokines’ release, namely, tumor necrosis factor-α (TNF-α), interleukin-1β (IL-1β), and IL-6 (Ji et al., 2009). It clearly indicates that agonist activity at PPAR-γ receptor is beneficial in neurodegenerative disorders to control neuroinflammation, oxidative stress, and mitochondrial dysfunction. In this backdrop, we have designed and selected two novel glitazones from an in-house library of glitazones based on their docking scores against PPAR-γ protein and results of *in silico* computational pharmacoinformatics and toxicity studies. The glitazones, namely, G1 and G2 (Figure 1), have been subjected for the neuroprotective evaluation in intracerebroventricular administration of lipopolysaccharide (ICV-LPS) neuroinflammatory model in rats. The results of the novel glitazones in each parameter of this study were compared with standard pioglitazone to understand the therapeutic efficacy and possible mechanism of the developed novel glitazones.

## Materials and Methods

### Molecular Docking Studies

To gain a more meaningful understanding of protein binding of the embarked ligands G1 and G2, we have performed the molecular docking studies by using Surflex-Dock (Jain, 2003, 2007; Haider et al., 2014; Singh et al., 2018) application available with SYBYL-X 2.1.1 Software package. The target protein (PPAR-γ bound to PGC-1α; PDB Code: 3CS8) (Li et al., 2008) was sourced from PDB^1^. The crystal structure of the target protein was prepared successively by removing the water molecules, adding hydrogen, building the missing residues, and fixing the protonation state. Energy minimization protocol was also applied by adopting the Powell method (Fletcher and Powell, 1963; Nelder and Mead, 1965; Spałek et al., 2005) using Gasteiger–Marsili (Meng et al., 1992; Kim and Greco, 1998) charges and MMFF94s force field (Halgren, 1995) available with SYBYL-X 2.1.1 software package (Spitzer and Jain, 2012). Equitably, synthesized ligands G1 and G2 were also sketched and prepared by applying Gasteiger–Huckel (Dixit et al., 2004) charges and MMFF94s force field using the same SYBYL-X 2.1.1 software package. Consecutively, the energy and parameter optimized files of protein and ligand were then intermingled by using Surflex Dock application to find docking interactions. Analysis of the adopted docking protocol was carried out using the same program, and binding poses of the synthesized ligands along with the reference compound to the active site of the protein were analyzed and compared. The key amino acid residues at the active site that are involved in the hydrogen bonds are listed with the distance. The different scores of this molecular docking study are described below for easier understanding. *Total Score*: Total score is summative of the degree of appropriate penetration of ligand into the target protein during the process of docking. A higher total score indicates significant binding interactions. *Crash Score*: The crash score is the degree of inappropriate penetration into the protein by the ligand as well as the degree of internal self-clashing that the ligand is experiencing. Crash scores that are close to 0.0 are favorable. *Polar Score*: Contribution of the polar non-hydrogen bonding interactions to the total score. The polar score may be useful for excluding docking results that make no hydrogen bonds. *Chem Score*: Points for H-bonding, lipophilic contact, and rotational entropy, along with an intercept term.

### Computational Pharmacoinformatics Studies (ADME, Drug Likeness, and Toxicity Predictions)

The designed novel glitazones were submitted to ADMET and TOPKAT tools of small-molecule protocol implemented in the Discovery studio 2020 to assess and predict the *in silico* pharmacokinetic parameters. Pharmacoinformatic parameters such as human intestinal absorption (HIA), aqueous solubility, blood–brain barrier (BBB) penetration, cytochrome CYP2D6 inhibition, plasma protein binding (PPB), and hepatotoxicity were assessed. The toxicity study included NTP rodent carcinogenicity and Ames mutagenicity animal models. These models were developed and validated based on quantitative structure toxicity relationship (QSTR) principle (Veber et al., 2002; Lipinski, 2004).

### Acute Toxicity and Pharmacological Evaluation

#### Chemicals

Lipopolysaccharides was obtained from Sigma-Aldrich, Bengaluru, India. TNF-α, IL-1β, and IL-6 ELISA kits were obtained from Invitrogen, Thermo Fisher Scientific, Waltham, MA, United States. Thiobarbituric acid (TBA), sulfanilamide, sulfosalicylic acid (SSA), and Tris–HCl were procured from HiMedia, Mumbai, India. Phenazonium methosulfate (PMS), nicotinamide adenine dinucleotide reduced (NADH), and butylated hydroxyl toluene (BHT) were obtained from Sisco Research Laboratories, Chennai, India. All other chemicals and solvents used in this study were of analytical grade.

#### Experimental Animals

The experimental animals were supplied from the central animal house facility, Department of Pharmacology, JSS College of Pharmacy, Ooty, Tamil Nadu, India. The housing of experimental animals and all the experimental procedures were carried out according to the guidelines (Guide for the Care and Use of Laboratory Animals) prescribed by the Indian Council of Medical Research, New Delhi, India. Animals were accustomed to the experimental room 2 weeks prior to the designed experiment. Animals were quarantined under precise conditions such as temperature (22 ± 3°C) and humidity at 40–60%. The rats were kept in spaced cages during the experimental period and fed with water and pellet food *ad libitum*. The study protocol has been approved (Approval No: JSSCP/IAEC/M.Pharm/Pharmacology/01/2016-2017) by the Institutional Animal Ethical Committee (IAEC), JSS College of Pharmacy, Ooty, Tamil Nadu, India.

#### Acute Toxicity Study

Acute oral toxicity study for novel glitazones was conducted as per OECD 423 guidelines. The compounds (G1 and G2) were tested in the following doses: 5, 50, 300, and 2000 mg/kg. Each group was consisted with six Wistar rats weighing 180–200 g (three male and three female) and a total of eight groups were assigned (G1: 5, 50, 300, and 2000 mg/kg; G2: 5, 50, 300, and 2000 mg/kg). The study started from a small dose (5 mg/kg) to a large dose (2000 mg/kg) per OECD 423 guidelines. After administration of test compounds, rats were observed for clinical signs and mortality at 0, 0.5, 1, 2, 4, 8, 12, and 24 h, and the same observation continued for every 24 h until 14 days. Parameters like hyperactivity, twitching, piloerection, irritability, rigidity, jumping, convulsions, ptosis, sedation, and sleep loss were observed during the study period.

#### Neuroprotective Evaluation

Six-month-old male Wistar rats weighing 200–250 g were used for the neuroprotective evaluation. Animals were divided into seven groups, and each group consisted of nine animals. All the nine animals per group were subjected to behavior studies. After behavior assessments, three animals were used for antioxidant parameters [LPO, superoxide dismutase (SOD), catalase (CAT), glutathione (GSH), and NO] evaluation. Three animals were used for pro-inflammatory cytokines (TNF-α, IL-1β, and IL-6) estimation and another three were used for histopathological studies. Groups were assigned as follows: Group 1 – Sham Operated (SO), Group 2 – Lipopolysaccharide (2 μg/μl) (LPS), Group 3 – LPS + Pioglitazone (20 mg/kg) (Pio), Group 4 – LPS + Glitazone 1 (15 mg/kg) (G1-15), Group 5 – LPS + Glitazone 1 (30 mg/kg) (G1-30), Group 6 – LPS + Glitazone 2 (15 mg/kg) (G2-15), and Group 7 – LPS + Glitazone 2 (30 mg/kg) (G2-30).

### ICV Administration of LPS – Induction of Neuroinflammation

Rats were anesthetized using intraperitoneal administration of ketamine (80 mg/kg) and xylazine (10 mg/kg). After anesthesia, ICV administration of LPS was done by using the stereotaxic apparatus. The head of rat was positioned exactly in an apparatus frame and a midline sagittal incision was done in the scalp. A burr hole was drilled on the fourth ventricle under the following coordinates: 2.5 mm posterior from lambda, on the midline, 7 mm below the dura. Then, the LPS (2 μg/1 μl) was dissolved in artificial cerebrospinal fluid (aCSF – 147 mM NaCl, 2.9 mM KCl, 1.6 mM MgCl_2_, 1.7 mM CaCl_2_, and 2.2 mM dextrose) and slowly injected via ICV using a Hamilton micro-syringe at an infusion rate of 1 μl/min. The total volume of LPS administered was 5 μl per animal. Post-operative care encompassed neomycin topical ointment applied to the exposed skull and scalp prior to closure. Lidocaine was rubbed locally to the scalp to reduce the pain, and 5 ml of sterile isotonic saline was injected subcutaneously to prevent the dehydration during recovery. The rats were closely monitored until recovery and bodyweight and temperature were monitored periodically (Joshi et al., 2014; Prathab Balaji et al., 2015). Prior to induction of neuroinflammation, the treatments with glitazones (G1 and G2) and standard pioglitazone were made for four consecutive days to respective groups. On the fifth day, neuroinflammation was induced in all the six groups except the sham-operated group through ICV administration of LPS. After 7 days, the animals were subjected to behavior assessments and the animals were sacrificed for neurobiochemical and inflammatory cytokine estimation followed by histopathological studies.

### Behavioral Tests

#### Actophotometer Test

The motor coordination was assessed by keeping the animals in an actophotometer for 6 min. The body movement of the animal cuts off a beam of light falling in the path of a photocell, and count is recorded and displayed digitally. The count indicates the number of ambulation of the animals during the 5-min duration (Prathab Balaji et al., 2015).

#### Rotarod Test

The rotarod apparatus was turned on and 20 rpm was then selected as an appropriate speed. Each rat was given five trials before the real reading was recorded. The animal was placed individually one by one on the rotating rod. The “fall-off time” was noted when the animal falls from the rotating rod, and the fall-off time of the normal group was compared with that of the treatment group (Balkaya et al., 2013).

### Neurobiochemical Evaluation

#### Brain Sample Preparation

After completion of behavioral assessments, the rats were sacrificed by excessive anesthesia. Then, the brain samples were quickly isolated and washed with chilled saline and stored at −80°C until further evaluations. The whole brain samples were homogenized with 10% ice-cold KCl (a quantity of 100 μl of KCl for a quantity of 10 mg of tissue) for the following neurobiochemical analysis.

#### Lipid Peroxide (LPO) Assay (Malondialdehyde Assay)

Lipid peroxidation was evaluated by measuring the TBAR content according to the TBA test described by Ohkawa et al. (1979) with slight modifications. The incubation mixture consists of 0.5 ml of aliquot, 0.2 ml of 8% sodium dodecyl sulfate, 1.5 ml of 0.9% aqueous solution of TBA, and double distilled water bath for 30 min. After cooling, the red chromogen was extracted into 5 ml of mixture of n-butanol and pyridine (15.1 v/v) centrifuged at 4000 rpm for 10 min. The absorbance of the organic layer was taken at 532 nm (UV, Shimadzu, Japan). 1,1,3,3-Tetra ethoxy propane was used as an external standard in the concentration range of 80–240 nmol (Ohkawa et al., 1979).

#### Superoxide Dismutase (SOD)

The sodium pyrophosphate buffer (0.025 M, pH 8.3) in a quantity of 0.3 ml was added to 0.05 ml of homogenate. To this mixture, 0.025 and 0.075 ml of PMS (186 μM) and NBT (300 μM in buffer, pH 8.3) were added. The initiation of the reaction was commenced by the instillation of 0.075 ml of NADH. The mixture was then incubated at a temperature of 30°C for a period of 90 s. 0.25 ml of glacial acetic acid was added in-order to arrest the ongoing reaction. N-butanol (2 ml) was shaken vigorously along with the reaction mixture; later, the mixture was centrifuged at 4000 rpm for 1 min. The colorimetric analysis was carried out at 560 nm using a spectrophotometer, with n-butanol (1.5 ml) serving as blank (Kakkar et al., 1984).

#### Catalase (CAT)

A small quantity of brain homogenate (100 μl) or sucrose (0.32 M) was subjected to incubation with potassium phosphate buffer (2.25 ml) 65 mM at pH 7.8 for 30 min at 25°C. The initiation of the reaction was by the addition of hydrogen peroxide (7.5 mM; 650 μl). The absorbance change was measured for a period of 2–3 min at 240 nm (UV, Shimadzu, Japan) (Beers and Sizer, 1952).

#### Reduced Glutathione (GSH)

Glutathione content was estimated by following the method of Jollow et al. (1974). 0.25 ml of brain homogenate was added to an equal volume of ice-cold 5% TCA. The precipitate was removed by centrifugation at 4000 rpm for 10 min. To a 1-ml aliquot of supernatant, 0.25 ml of 0.2 M phosphate buffer (pH 8.0) and 0.5 ml of DTNB (0.6 mM in 0.2 M phosphate buffer, pH 8.0) were added and mixed well. The absorbance was read at 412 nm using a spectrophotometer (UV, Shimadzu, Japan) (Jollow et al., 1974).

#### NO Assay

Nitric oxide was indirectly measured in the form of nitrates and nitrites taking 0.2 ml of 10% homogenate followed by the addition of 1.8 ml of saline and 0.4 ml of 35% sulfosalicylic acid for protein precipitation. The precipitate was removed by centrifugation at 4000 rpm for 10 min. To a 1-ml aliquot of supernatant, 2 ml of Griess reagent (1 g of sulfanilamide was dissolved in a small volume of water, to which 2 ml of orthophosphoric acid and 100 mg of naphthyl ethyl diamine were added, and the volume was made up to 100 ml) was added. The mixture was allowed to stand for 20 min under dark conditions. The color intensity was read at 540 nm (UV, Shimadzu, Japan). Standard calibration was plotted using sodium nitrite in the concentration range 200–1000 ng (Green et al., 1982).

### Pro-inflammatory Cytokines Estimation by ELISA

The brain samples were reconstituted with buffer (0.1% BSA, 81 mM Na_2_HPO_4_, 19 mM NaH_2_PO_4_, 50 mM NaCl, and 0.1% Triton X-100, pH 7.4), and the level of pro-inflammatory cytokines like TNF-α, IL-1β, and IL-6 was quantified using an ELISA kit (Invitrogen, United States) as per the manufacturer’s instruction (Mannan Thodukayil et al., 2019).

### Histopathology – Hematoxylin and Eosin Staining

The brain samples were submerged in formalin for fixation and soaked in alcohol to remove the lipid debris. Then, brain samples were fixed in paraffin wax and 5-μm coronal sections were obtained in the prefrontal cortex region of the brain. Processing of the sections was done followed by staining with hematoxylin and eosin (H&E). Ethanol was used for dehydrating the sections. Thereafter, the sections were microscopically observed under 40× objective, and the plexiform layer of the cortical tissue region of the brain was photographed in order to understand the extension of neuroprotection (Aras et al., 2015). The histopathological changes were observed and percentage of neuronal damage was quantified using ImageJ software.

### Statistical Analysis

The values were expressed as the mean ± SEM. All the data were statistically analyzed by using GraphPad Prism 6.0 software. Statistical significance was determined by one-way ANOVA followed by Bonferroni’s Multiple Comparison Test to assess differences between the groups. Values were considered statistically significant if *p* < 0.05.

## Results

### Molecular Docking

Docking results in the form of Total Score and Chem Score are shown in Table 1. The binding modes of glitazones with target protein (PDB Code: 3CS8) are compared with the standard pioglitazone. Results of docking run explored the relatively good binding affinities of glitazones and further equated with respect to Total Score and Chem Score. The Total Score of the reference ligand pioglitazone and G1 was found to be higher and similar, indicating better binding with PPAR-γ. However, the Total Score of G2 was found to be relatively less. The Chem Score of G1 was observed to be highest even when compared to pioglitazone. The Crash Score and Polar Score were topped by pioglitazone, whereas G1 and G2 showed similar scores. Altogether, it was observed that G1 and G2 have shown similar docking results to pioglitazone. This prompted us to select these two glitazones for further experimental neuroprotective evaluation studies.

**TABLE 1:** *In sillco* docking results of novel glitazones (G1 & G2) and reference ligand pioglitazone.

Moleule	Total score	Chem score	Crash	Polar
Pioglitazone	8.3676	−17.6627	−3.0248	5.7878
G1	8.0068	−21.769	−1.296	1.1187
G2	7.2532	−17.9346	−1.2707	1.8716

**FIGURE 2:**
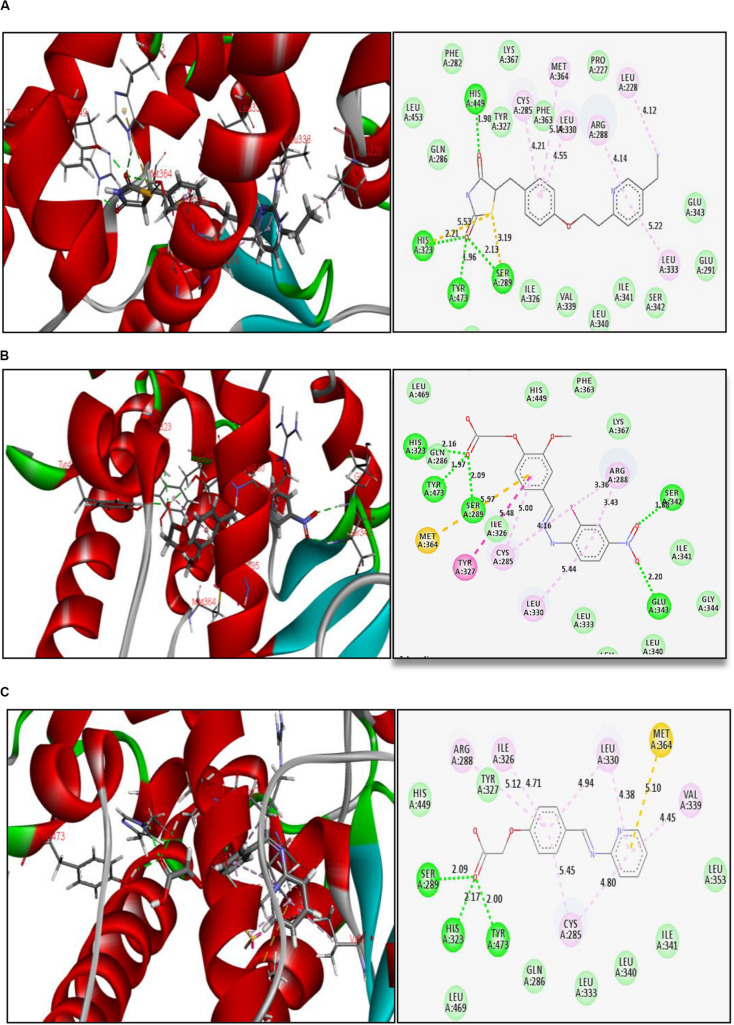
Binding interactions of **(A)** reference ligand pioglitazone, **(B)** glitazone 1 (G1), and **(C)** glitazone 2 (G2) at the active site of PPAR-γ, with green lines indicating strong H-bond, yellow lines indicating Π–sulfur bonds, and purple lines indicating hydrophobic interactions.

The reference ligand (pioglitazone) forms H-bond interactions with amino acid residues at the active site of PPAR-γ. The key residues are His 449, His 323, Tyr 473, and Ser 289, with H-bond distances ranging from 1.90 to 2.21 Å, holding the ligand in the binding pocket. Other interactions were also observed like Π–sulfur bond and Π–Π hydrophobic interactions (Figure 2A). Thiazolidinedione moiety of reference ligand (pioglitazone) makes some important interactions, and this is attributed to the carbonyl groups of the thiazolidinedione scaffold.

Interestingly, the glitazones G1 and G2 also showed similar binding interactions to amino acid-like standards. The interacting amino acids were His 323, Tyr 473, Ser 289, Ser 242, and Glu 343, with H-bond distances ranging between 1.88 and 2.20 Å, confirming stable complexes (Figures 2B,C). Other associative bonds like Π–sulfur and Π–Π interactions were also observed in the complexes. The common binding functional moiety oxyacetic acid of G1 and G2 bonded to the active site of protein via H-bonding. The population of varied intermolecular interactions formed in the complexes of target protein with reference (pioglitazone); G1 and G2 indicate the similar biological activity of all the three candidates.

### Computational Pharmacoinformatics Studies

**FIGURE 3:**
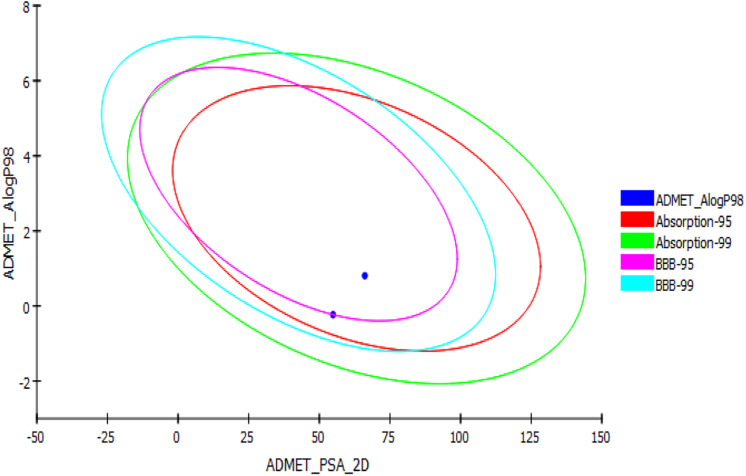
ADMET plot for glitazones: Plot indicates that both the glitazones being inside the ellipses’ boundaries possess ideal ADMET properties.

*In silico* ADMET, TOPKAT, and drug likeness predictions for the two synthesized glitazones were performed to understand the possible insight of quantitative structure–property relations (QSPR). The specific models used in these *in silico* pharmacokinetics predictions were originally obtained from quantitative structure–activity relationship (QSAR) on a series of compounds. ADMET is used as first protocol to screen molecules. The important descriptors such as BBB penetration, hepatotoxicity, and CYP2D6 enzyme inhibition were assessed. In addition, Ames mutagenicity and carcinogenicity potential were predicted as toxicity measures. The results of the pharmacokinetic analysis are shown in Figure 3. The regular ADMET plot Alogp_98 vs. PSA presents Glitazone 1 being at the center of the plot, which indicates ideal properties such as good solubility, absorption, permeability, less hepatotoxicity, and better BBB permeation (95 and 99, based on measurement confidence interval).

Glitazone 2 also shows ideal properties that are within boundary ellipses of the pharmacoinformatic parameters measured but are less impressive when compared to Glitazone 1. Altogether, these two glitazones are predicted to be non-toxic to hepatic cells, to have very high to medium BBB permeation, and to be a non-inhibitor of a metabolic enzyme (CPYD26) (Table 2). TOPKAT models for toxicity have shown that these glitazones (G1 and G2) are free from carcinogen and mutagen potentials. QSTR-based toxicity prediction parameters are shown in Table 2. For drug likeness, we checked these glitazones for Lipinski’s RO5 violations [molecular weight ≤500, log *p* ≤ 5, hydrogen bond acceptors (HBA) ≤10, and hydrogen bond donor (HBD) ≤5]. However, both the glitazones did not show any violations, except for Glitazone 2, which showed HBA as 12 (Table 3).

**TABLE 2:** *In sillco* ADME properties and TOPKAT toxicity model analysis of novel glitazones.

Glitazone	BBB	Solubility	CPY2D6	Hepatotoxic	HIA	Alogp_98	PSA	TOPKAT	
								Ames mutagen	NTP carcinogen
G1	3	3	NI	NT	0	2.251	87.031	NC	NM
G2	3	3	NI	NT	0	2.987	92.086	NC	NM

*NI, non-inhibitor; NT, non-toxic; NC, non-carcinogen; NM, non-mutagen.*

**TABLE 3:** *In sillco* drug likeness analysis of novel glitazones.

Glitazones	Lipinski’s rule of 5 parameters			
	HBD	HBA	MW	logP
G1	1	5	256.08	2.42
G2	2	12	390.08	2.76

*HBD, hydrogen bond donor; HBA, hydrogen bond acceptor; MW, molecular weight; logP, partition coefficient.*

### Acute Oral Toxicity Study

The novel glitazones were subjected to acute toxicity studies as per the OECD 423 guidelines to assess the toxicity profile. Since there were no toxicity signs such as hyperactivity, irritability, convulsions, huge weight change, and mortality at the dose of 5 and 50 mg/kg, the acute toxicity studies were conducted with 300 and 2000 mg/kg. The acute toxicity studies showed that glitazones exhibit some toxicity signs like hyperactivity, irritability, convulsions, and huge weight change, and two mortalities were observed at the dose of 2000 mg/kg, whereas in the dose of 300 mg/kg, glitazones did not produce any significant toxicity signs and mortality. Hence, further neuroprotective evaluations with novel synthesized glitazones have been conducted with 1/10 and 1/20 of the 300 mg/kg, i.e., 30 and 15 mg/kg, respectively.

### Neurobehavioral Studies

#### Actophotometer Test

**FIGURE 4:**
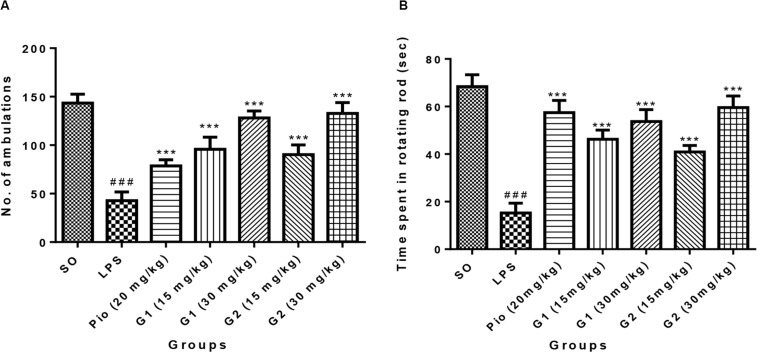
Effect of novel glitazones on **(A)** actophotometer test and **(B)** rotarod test of ICV-LPS-administered rats. Values are expressed as mean ± SEM. Statistical significance was determined by one-way ANOVA followed by Bonferroni’s multiple comparison tests. Superscript ### denotes *p* < 0.001 vs. SO and *** denotes *p* < 0.001 vs. LPS. SO, sham operated; LPS, lipopolysaccharide; Pio, pioglitazone; G, glitazones.

Intracerebroventricular administration of LPS in rats has significantly decreased the number of ambulations [*F*_(__9_,_56__)_ = 22.33; *p* < 0.0001], indicating that LPS-induced neuroinflammation has reduced the motor coordination of animals. Treatment with glitazones G1 (15 mg/kg) [*F*_(__9_,_56__)_ = 11.77; *p* < 0.0001], G1 (30 mg/kg) [*F*_(__9_,_56__)_ = 18.91; *p* < 0.0001], G2 (15 mg/kg) [*F*_(__9_,_56__)_ = 10.54; *p* < 0.0001], and G2 (30 mg/kg) [*F*_(__9_,_56__)_ = 19.97; *p* < 0.0001] significantly increased the number of ambulations, showing that glitazones have attenuated the intensity of neuroinflammation and improved the motor coordination of animals. Interestingly, the results were similar with standard pioglitazone-treated groups [*F*_(__9_,_56__)_ = 7.953; *p* < 0.0001], which is shown in Figure 4A.

#### Rotarod Test

The group administered with LPS through ICV have remarkably decreased [*F*_(__9_,_44__)_ = 25.17; *p* < 0.0001] the time spent by rats in the rotating rod, indicating that treatment with LPS has reduced the muscle coordination of the animals through inducing neuroinflammation. The administration of novel glitazones G1 (15 mg/kg) [*F*_(__9_,_44__)_ = 14.69; *p* < 0.0001], G1 (30 mg/kg) [*F*_(__9_,_44__)_ = 18.22; *p* < 0.0001], G2 (15 mg/kg) [*F*_(__9_,_44__)_ = 12.16; *p* < 0.0001], and G2 (30 mg/kg) [*F*_(__9_,_44__)_ = 21.01; *p* < 0.0001] have significantly increased the muscle coordination of the LPS-administered animals, which is characterized by the animals that have spent more time in the rotating rod. The effect of novel glitazones on the rotarod test of the LPS-administered rats is comparable [*F*_(__9_,_44__)_ = 20.01; *p* < 0.0001] with standard treatment of pioglitazone (Figure 4B).

### Neurobiochemical Evaluation

#### Malondialdehyde (Lipid Peroxidation)

**FIGURE 5:**
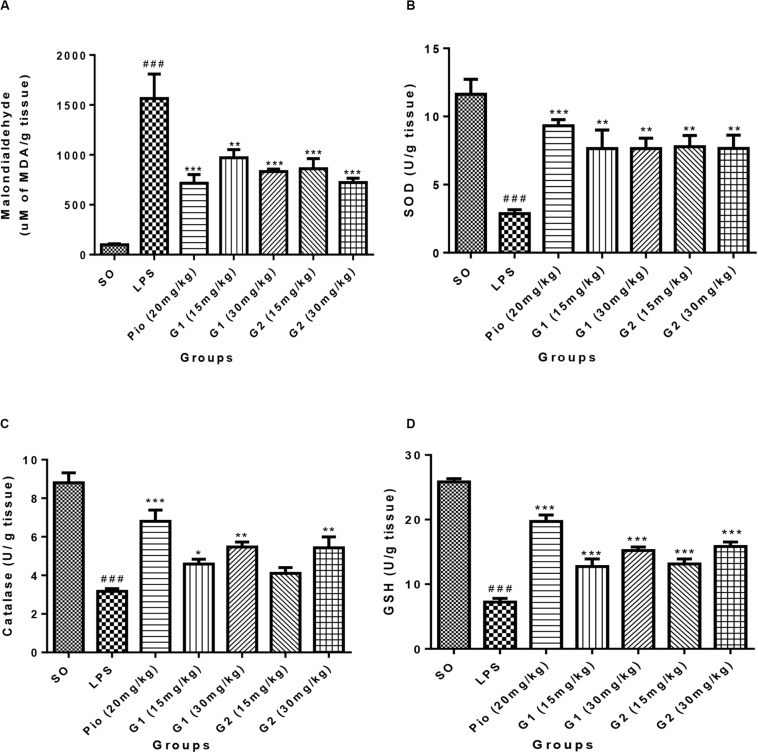
Effect of novel glitazones on brain **(A)** malondialdehyde (LPO), **(B)** SOD, **(C)** CAT, and **(D)** GSH level in ICV-LPS-administered rats. Values are expressed as mean ± SEM. Statistical significance was determined by one-way ANOVA followed by Bonferroni’s multiple comparison tests. Superscript ### denotes *p* < 0.001 vs. SO; *, **, *** denote *p* < 0.05, *p* < 0.01, *p* < 0.001 vs. LPS, respectively. SO, sham operated; LPS, lipopolysaccharide; Pio, pioglitazone; G, glitazones.

The level of malondialdehyde is significantly increased [*F*_(__3_,_14__)_ = 16.07; *p* < 0.0001] in the brain of LPS-administered rats, indicating enhanced lipid peroxidation in neurons due to release of oxy-radicals after LPS administration, which is summarized in Figure 5A. The novel glitazones G1 (15 mg/kg) [*F*_(__3_,_14__)_ = 6.519; *p* = 0.0003], G1 (30 mg/kg) [*F*_(__3_,_14__)_ = 8.034; *p* < 0.0001], G2 (15 mg/kg) [*F*_(__3_,_14__)_ = 7.717; *p* < 0.0001], and G2 (30 mg/kg) [*F*_(__3_,_14__)_ = 9.235; *p* < 0.0001] administration in LPS-treated rats have shown decreased level of malondialdehyde, indicating that glitazones have scavenged the radical release after LPS administration. The standard pioglitazone [*F*_(__3_,_14__)_ = 9.310; *p* < 0.0001] administration also reduced the level of brain malondialdehyde in LPS-administered rats, and it is comparable with the effect of novel glitazones during inflammatory conditions (Figure 5A).

#### Superoxide Dismutase (SOD)

The administration of LPS in rats through ICV has significantly depleted [*F*_(__3_,_14__)_ = 11.99; *p* < 0.0001] the SOD level in the brain in comparison to sham-operated rats. Treatment with pioglitazone (20 mg/kg) has increased [*F*_(__3_,_14__)_ = 8.831; *p* < 0.0001] the level of SOD in the brain of LPS-administered rats. Administration of glitazones G1 (15 mg/kg) [*F*_(__3_,_14__)_ = 6.541; *p* = 0.0003], G1 (30 mg/kg) [*F*_(__3_,_14__)_ = 6.537; *p* = 0.0003], G2 (15 mg/kg) [*F*_(__3_,_14__)_ = 6.730; *p* = 0.0002], and G2 (30 mg/kg) [*F*_(__3_,_14__)_ = 6.554; *p* = 0.0003] have significantly (*p* < 0.001) increased the SOD level in the brain of LPS-administered rats, and the results were similar to standard pioglitazone, which is depicted in Figure 5B.

#### Catalase (CAT)

The level of CAT in the brain of LPS-infused rats has significantly decreased [*F*_(__3_,_14__)_ = 16.86; *p* < 0.0001] in comparison to SO rats, which are represented in Figure 5C. The increased level of brain CAT was observed in rats treated with G1 (15 mg/kg) [*F*_(__3_,_14__)_ = 4.250; *p* = 0.0170], G1 (30 mg/kg) [*F*_(__3_,_14__)_ = 6.900; *p* = 0.0002], and G2 (30 mg/kg) [*F*_(__3_,_14__)_ = 6.764; *p* = 0.0002]. The treatment with standard pioglitazone rats has shown significant elevation of brain CAT level (*p* < 0.001) [*F*_(__3_,_14__)_ = 10.88; *p* < 0.0001], which is comparable with test glitazones.

#### Reduced Glutathione (GSH)

The GSH level in the brain was significantly [*F*_(__3_,_14__)_ = 28.77; *p* < 0.0001] reduced after administration of LPS through ICV in comparison to SO rats. The treatment with pioglitazone remarkably increased the brain GSH level [*F*_(__3_,_14__)_ = 19.30; *p* < 0.0001] in LPS-administered rats, and similar results were obtained with G1 (15 mg/kg) [*F*_(__3_,_14__)_ = 8.487; *p* < 0.0001], G1 (30 mg/kg) [*F*_(__3_,_14__)_ = 12.33; *p* < 0.0001], G2 (15 mg/kg) [*F*_(__3_,_14__)_ = 9.123; *p* < 0.0001], and G2 (30 mg/kg) [*F*_(__3_,_14__)_ = 13.30; *p* < 0.0001] treatments, which are summarized in Figure 5D.

#### Nitrite/Nitrate (NO)

**FIGURE 6:**
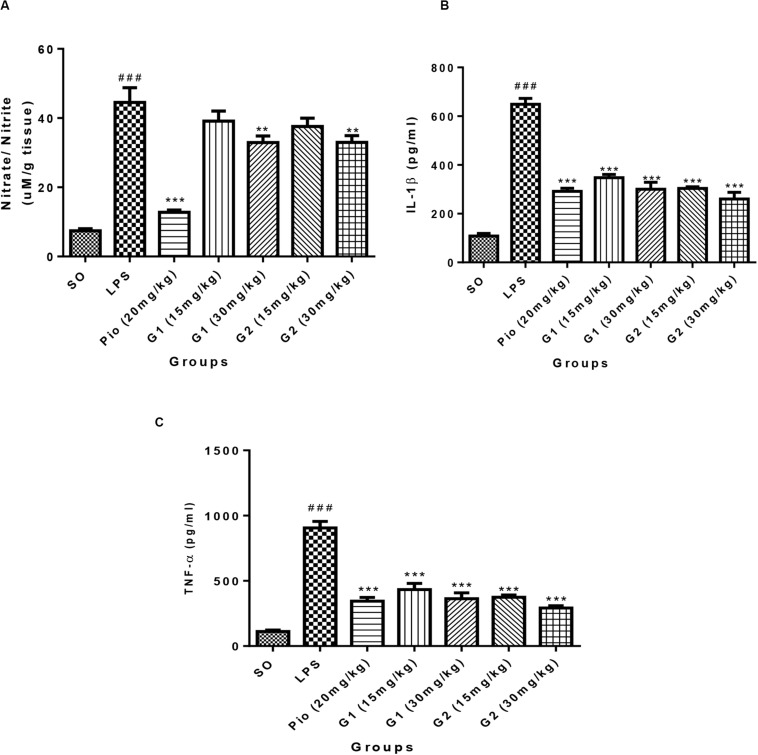
Effect of novel glitazones on brain **(A)** NO, **(B)** IL-1β, and **(C)** TNF-α level in ICV-LPS-administered rats. Values are expressed as mean ± SEM. Statistical significance was determined by one-way ANOVA followed by Bonferroni’s multiple comparison tests. Superscript ### denotes *p* < 0.001 vs. SO; ** and *** denote *p* < 0.01 and *p* < 0.001 vs. LPS, respectively. SO, sham operated; LPS, lipopolysaccharide; Pio, pioglitazone; G, glitazones.

Lipopolysaccharides administration in rats through ICV has increased [*F*_(__3_,_14__)_ = 18.71; *p* < 0.0001] the NO level in the brain in comparison to SO rats, which is presented in Figure 6A. The level of NO in the brain was significantly [*F*_(__3_,_14__)_ = 15.83; *p* < 0.0001] reduced after treatment with standard pioglitazone. The G1 (30 mg/kg) [*F*_(__3_,_14__)_ = 5.642; *p* = 0.0013] and G2 (30 mg/kg) [*F*_(__3_,_14__)_ = 5.177; *p* = 0.0029] treatments have reduced the brain NO level in LPS-administered rats like pioglitazone treatment.

### Pro-inflammatory Cytokines

#### IL-1β

Administration of LPS in rats through ICV has significantly elevated the level of brain cytokine IL-1β [*F*_(__3_,_14__)_ = 34.22; *p* < 0.0001] in comparison to SO rats, which is summarized in Figure 6B. The level of IL-1β in the brain has significantly reduced after treatment with standard pioglitazone [*F*_(__3_,_14__)_ = 22.61; *p* < 0.0001]. The treatment with glitazones, G1 (15 mg/kg) [*F*_(__3_,_14__)_ = 19.07; *p* < 0.0001], G1 (30 mg/kg) [*F*_(__3_,_14__)_ = 22.07; *p* < 0.0001], G2 (15 mg/kg) [*F*_(__3_,_14__)_ = 21.88; *p* < 0.0001], and G2 (30 mg/kg) [*F*_(__3_,_14__)_ = 24.58; *p* < 0.0001] have significantly reduced the brain IL-1β level in LPS-administered rats. The effect of novel glitazones on brain IL-1β level in LPS-administered rats is similar and comparable with standard pioglitazone treatment.

#### TNF-α

The level of TNF-α is significantly increased [*F*_(__3_,_14__)_ = 28.22; *p* < 0.0001] in the brain of LPS-administered rats, indicating the intensity of neuroinflammation after LPS administration, which is summarized in Figure 6C. The novel glitazones G1 (15 mg/kg) [*F*_(__3_,_14__)_ = 16.83; *p* < 0.0001], G1 (30 mg/kg) [*F*_(__3_,_14__)_ = 19.28; *p* < 0.0001], G2 (15 mg/kg) [*F*_(__3_,_14__)_ = 18.91; *p* < 0.0001], and G2 (30 mg/kg) [*F*_(__3_,_14__)_ = 21.81; *p* < 0.0001] administration in LPS-treated rats have shown a decreased level of TNF-α, denoting that glitazones have ameliorated the cytokine release after LPS administration. The standard pioglitazone [*F*_(__3_,_14__)_ = 19.99; *p* < 0.0001] administration also reduced the level of brain TNF-α in LPS-administered rats, and it is comparable with the effect of novel glitazones during inflammatory conditions.

### Histopathological Studies

#### H&E Staining

**FIGURE 7:**
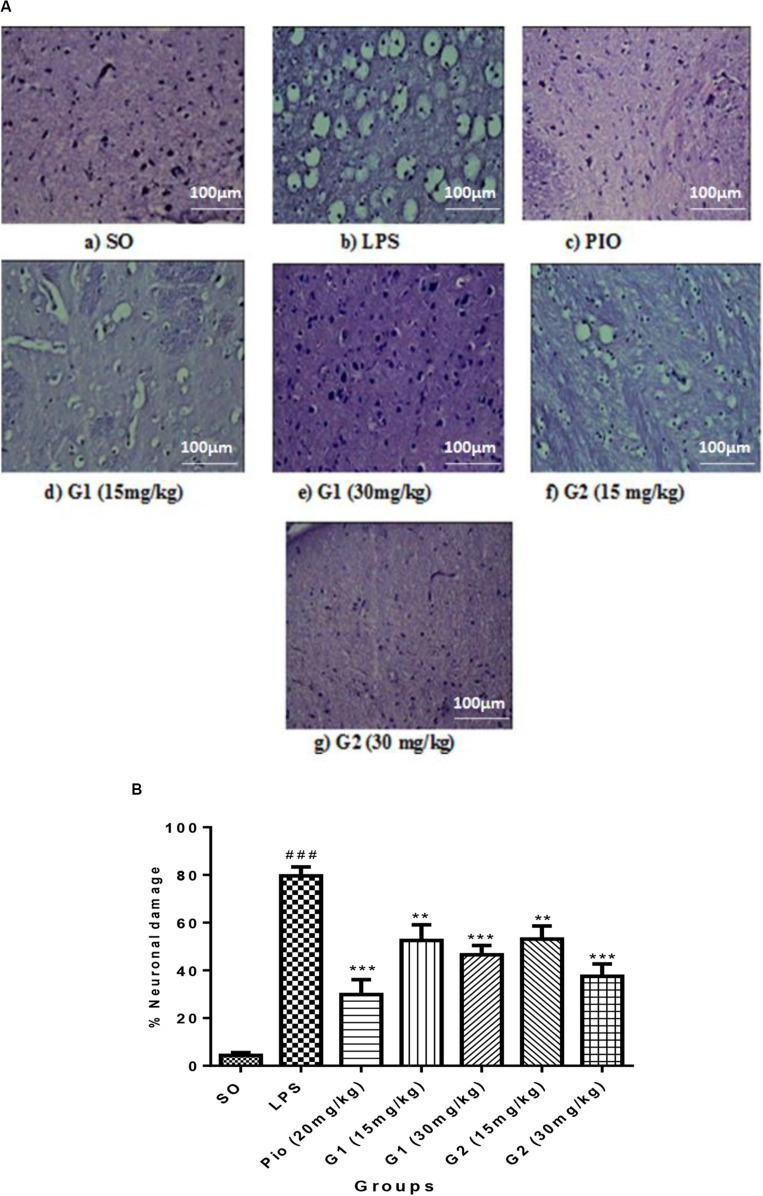
**(A)** Sections of the cortex region of the brain stained with hematoxylin and eosin staining. **(B)** Quantification of neuronal damage. Values are expressed as mean ± SEM. Statistical significance was determined by one-way ANOVA followed by Bonferroni’s multiple comparison tests. Superscript ### denotes *p* < 0.001 vs. SO; ** and *** denote *p* < 0.01 and *p* < 0.001 vs. LPS, respectively. SO, sham operated; LPS, lipopolysaccharide; Pio, pioglitazone; G, glitazones.

The histopathology studies demonstrate that the cortex region of brain tissue of the SO group remains intact, the cell organelles and neurons were kept well-arranged, and the nuclei were centered with clear staining. LPS administration through ICV has shown severe vacuolization and edema were observed. Neurons were markedly degenerated and became necrotic, and their arrangement was disordered with LPS administration. However, pioglitazone (20 mg/kg) and glitazone G1 and G2 (30 and 15 mg/kg) groups have shown that the extent of damage was significantly diminished, with decreased vacuolization and decreased neuronal degeneration that are characterized by less edema and swelling of neurons with intact cells (Figure 7A).

The percentage of neuronal damage was significantly increased [*F*_(__3_,_14__)_ = 18.66; *p* < 0.0001] in ICV-LPS-administered rats, evidencing the neuroinflammation-mediated neuronal death. Treatment with standard pioglitazone significantly decreased [*F*_(__3_,_14__)_ = 12.34; *p* < 0.0001] the percentage neuronal damage. Interestingly, administration of novel glitazones G1 (15 mg/kg) [*F*_(__3_,_14__)_ = 6.723; *p* = 0.0002], G1 (30 mg/kg) [*F*_(__3_,_14__)_ = 8.201; *p* < 0.0001], G2 (15 mg/kg) [*F*_(__3_,_14__)_ = 6.576; *p* = 0.0003], and G2 (30 mg/kg) [*F*_(__3_,_14__)_ = 10.44; *p* < 0.0001] has remarkably decreased the percentage of neuronal damage in LPS-administered rats, and the effects were comparable with the standard drug pioglitazone (Figure 7B).

## Discussion

Neuroinflammation plays an imperative role in several neurodegenerative diseases such as AD, MS, PD, amyotrophic lateral sclerosis (ALS), and cerebral ischemia (Chen et al., 2016). During neuroinflammatory conditions, the pathological features involved like permeability of BBB, destruction of myelin sheath, damage of axon, formation of glial scar, and the presence of inflammatory cells, mostly lymphocytes, infiltrating the central nervous system (Niranjan, 2018) resulted in severe neurodegeneration. It clearly emphasizes that inflammation in neuronal cells is a major cause of neurodegeneration, and therefore, targeting neuroinflammation is an effective strategy for the management of neurodegenerative disorders.

Recently, researchers are focusing on activation of PPAR-γ receptors to control the neuroinflammation in various neurodegenerative disorders due to its regulation of multiple genes involved in central inflammatory cascades (Villapol, 2018). Interestingly, glitazone types of chemical molecules that are used in the clinical treatment of diabetes mellitus were found to have anti-neuroinflammatory action and neuroprotection through central PPAR-γ agonism (Swanson et al., 2011). Though existing glitazones have exerted neuroprotection, their applicability in various neurodegenerative disorders is still challenging due to their unwanted effects (Mehtälä et al., 2018). In this backdrop, we have developed novel glitazones (G1 and G2) mainly emphasizing on favorable drug likeness, ADME, and toxicity properties while designing without affecting therapeutic effect.

The binding pattern of novel glitazones with target protein (PDB Code: 3CS8) is comparable with standard pioglitazone, and it has similar and equivalent Total Score and Chem Score in docking studies. Pioglitazone forms H-bond interactions (distance range 1.90 to 2.21 Å) in the active binding pocket of PPAR-γ protein with amino acid residues like His 449, His 323, Tyr 473, and Ser 289. In addition, Π–sulfur bond and Π−Π hydrophobic interactions were also observed. Interestingly, the glitazones G1 and G2 also have shown similar binding interactions like pioglitazone as the interacting amino acids were His 323, Tyr 473, Ser 289, Ser 242, and Glu 343, with H-bond (1.88–2.20 Å), ensuring the stable complexes (Justin et al., 2020). Also, the associative bonds like Π–sulfur and Π–Π interactions were also observed in the protein–ligand complexes and the common binding functional moiety oxyacetic acid of G1 and G2 have bonded with active binding pocket of protein through H-bonding. The population of diverse intermolecular interactions formed in the complexes of target protein with G1 and G2 ensures and represents a similar and better biological activity like the reference ligand pioglitazone.

To emphasize the drug likeness, ADME, and toxicity properties of the designed glitazones, we have performed *in silico* computational pharmacoinformatics studies. The ADMET plot Alogp_98 vs. PSA denotes that both glitazones have shown ideal properties such as good solubility, absorption, permeability, less hepatotoxicity, and better BBB permeation. In addition, two glitazones were predicted to be non-toxic to hepatic cells, to have very high to medium BBB permeation, and to be a non-inhibitor of a metabolic enzyme (CPYD26), which favors the pharmacokinetic properties of the desired glitazones. TOPKAT models for toxicity have shown that these glitazones (G1 and G2) are free from carcinogen and mutagen potential while the carcinogenicity is the serious adverse effect with existing clinical glitazones (Mehtälä et al., 2018). Lipinski’s RO5 assessment also ensures that both the glitazones did not violate the drug likeness properties. Altogether, the novel glitazones are safe and effective and have better desirable pharmacokinetic properties in computational studies; therefore, the above glitazones have been subjected to further evaluation using a suitable *in vivo* neuroinflammatory animal model as preliminary assessment.

The novel glitazones were subjected to acute toxicity studies as per the OECD 423 guidelines to assess the toxicity profile and to determine therapeutic range level. Since there were no toxicity signs such as hyperactivity, irritability, convulsions, huge weight change, and mortality at the dose of 5 and 50 mg/kg, the acute toxicity study was conducted with 300 and 2000 mg/kg. The acute toxicity studies have shown that glitazones exhibit some toxicity signs like hyperactivity, irritability, convulsions, and weight change, and two mortalities were observed at the dose of 2000 mg/kg, whereas at the dose of 300 mg/kg, glitazones did not produce any significant toxicity signs and mortality. Hence, further neuroprotective evaluations with novel synthesized glitazones have been conducted with 1/10 and 1/20 of the 300 mg/kg, i.e., 30 and 15 mg/kg, respectively.

Initially, the animals were pretreated with test and standard drugs and then neuroinflammation was induced by ICV administration of LPS dissolved in artificial CSF. The previous study suggests that ICV administration of LPS in rats serves as a good *in vivo* model mimicking the neuroinflammatory conditions to evaluate the neuroprotective activity of several agents (Prathab Balaji et al., 2015). Locomotor scores in the LPS group have been significantly reduced in comparison to SO rats, which indicates the impairment of motor coordination in rats. Studies have reported that ICV injection of LPS in rodents induces microglial activation, inflammatory cytokine release, and oxidative stress followed by dopaminergic neuronal death. The depletion of dopamine content in the retrorubral field, substantia nigra pars compacta (SNc), and ventral tegmental areas of the brain and dysfunctions of meso-striatal pathway in SNc together with cortico-striatal glutamatergic projections resulted in loss of motor coordination (Hoban et al., 2013). In the present study, LPS administration may lead to dysfunction of dopaminergic neurons by robust cytokines and radical release in meso-striatum and cortico-striatum, which results in motor inactivity in animals. Treatment with glitazones might have reduced cytokine release through PPAR-γ activation and thereby decreased the dopaminergic neuron degeneration in vital brain regions and increased the motor coordination of LPS-administered animals.

The rotarod experiment demonstrated impairment in muscle coordination in neuroinflammatory rats. An earlier report indicates that administration of LPS has exhibited poor performance of animals in the rotarod apparatus by inducing severe nigro-striatal lesion through triggering inflammatory cytokines and oxy-radicals (Liu et al., 2008; Choi et al., 2009). Treatment with glitazones has increased the rotarod performance of LPS-administered rats, evidencing that PPAR-γ-dependent regulation of cytokines and free radicals released by glitazones might have prevented the nigro-striatal lesion and increased the muscle coordination of LPS-infused animals.

It is a known fact that the reactive oxygen species play an important role in the pathogenesis of inflammation-induced neurotoxicity. Significant increase in the level of brain LPO was found in LPS-treated rats compared with normal rats, which are considered as an indication of oxidative stress and neuronal damage (Reed, 2011). The LPO is measured by thiobarbituric reactive substances, which is generally accepted as an indicator of oxidative stress (Ohkawa et al., 1979). In general, increased LPO level resulted in the loss of function and integrity of neuronal membranes, which later increases the non-specific permeability of ions leading to disruption of membrane structure (Shichiri, 2014). The treatment with pioglitazone (standard) and test glitazones decreased the LPO levels in LPS-administered brain, suggesting a protective effect of glitazones against LPS-induced oxidative stress. The antioxidant effect of novel glitazones might have been exerted through PPAR-γ activation. A previous report revealed that PPAR-γ activation with rosiglitazone has reduced hippocampal neuronal loss through enhancing the antioxidant activity. This neuroprotective effect was achieved by enhanced expression of SOD and GSH in hippocampus (Yu et al., 2008), which supports our present findings. The present research findings also show that treatment with novel glitazones has significantly increased the brain SOD, CAT, and GSH levels in LPS-infused animals. This PPAR-γ-dependent elevation of antioxidant enzymes by glitazones might reduce the LPO level and oxidative stress in neuroinflammatory conditions.

Activation of microglia by LPS through toll like receptor-4 (TLR-4) will increase the brain cytokines (TNF-α and IL-1β) and NO release (Ye et al., 2020). Since inflammation and elevated levels of nitrosative stress are associated with neurodegeneration, we have attempted to check the effect of novel glitazones on brain cytokines and NO levels. It has been reported that microglial cells present in the brain are the mainsource to release the cytokines and NO during neuroinflammation (Harry and Kraft, 2008). Treatment with standard and test glitazones decreased the cytokines (TNF-α and IL-1β) and NO level in the brain of LPS-administered rats, indicating that the glitazones might have inhibited the LPS-mediated microglial activation. This mechanism of glitazones may be attributed to PPAR-γ agonism because an earlier study showed that PPAR-γ agonists have been reported to control brain inflammation through controlling microglial activation (Bernardo and Minghetti, 2006). Histopathological studies also support our findings that treatment with glitazones has shown decreased vacuolization and neuronal degeneration that are characterized by less edema and swelling of neurons with intact cells. Interestingly, the observations of test glitazones are comparable with standard pioglitazone, and among the two glitazones, G2 has shown a significant effect compared to G1 in some of the parameters studied. The *in silico* findings also support that G2 has shown significant results in computational parameters when compared to G1. Though G2 exhibits better results, the neuroprotective efficacy of G1 cannot be ignored because G1 has also shown a similar effect like standard pioglitazones in most of the observations made it in the study.

Since the treatment with glitazones has shown a similar effect like that of the standard pioglitazone in most of the parameters of this study, the possible mechanism of neuroprotective effect of the test glitazones might be executed through activation of central PPAR-γ receptors. Consequently, PPAR-γ-dependent activation of PGC-1α signaling decreased the cytokines and free radicals released through regulating gene transcriptions. Previous studies stated that PGC-1α initiates neuroprotection through suppressing the ROS-mediated cell death (Rasbach and Schnellmann, 2007). PGC-1α also plays a central role by influencing the genes that regulate detoxification of ROS and inflammatory cytokines (Lu et al., 2010). It has also been considered that PGC-1α buffers oxidative stress by increasing the antioxidant enzymes SOD-1, SOD-2, catalase, and glutathione peroxidase-1 expression (St-Pierre et al., 2006). An earlier study also indicated that activation of PGC-1α could upregulate the expression of many target genes that are involved in neuronal survival and neuroprotection by inhibiting mitochondrial dysfunction, proteosomal dysfunction, oxidative stress, autophagy, neuroinflammation, and apoptosis (Rona-Voros and Weydt, 2010). Therefore, the developed novel glitazones exerted neuroprotection in neuroinflammation-induced rats by attenuation of brain inflammation and oxidative stress through activation of PGC-1α signaling via PPAR-γ agonism as shown in our *in silico* ligand–protein binding interaction studies.

## Conclusion

In the present study, activation of PGC-1α signaling by novel glitazones via PPAR-γ receptors might have regulated the genes associated with inflammation and oxidative stress in the neuron. The downregulation of inflammatory target proteins like NF-kB and upregulation of antioxidant enzymes resulted in decreased cytokines and free radicals released during neuroinflammatory conditions. The attenuation of brain cytokines and oxy-radical-mediated degeneration of neurons in vital motor regions like retrorubral field, SNc, nigro-striatal, and ventral tegmental would have reversed the behavioral dysfunction in an ICV-LPS neuroinflammatory animal model.

## Data Availability Statement

The datasets generated for this study are included in the article.

## Ethics Statement

The *in vivo* part of this study was reviewed and approved by the Institutional Animal Ethical Committee, JSS College of Pharmacy, Ooty, India (Approval No.: JSSCP/IAEC/M.Pharm/Pharmacology/01/2016-2017).

## Author Contributions

AJ: conceptualization. PA, JJ, VJ, and SD: methodology. CM, GB, and SY: investigation. SM and PP: software handling. BP: supervision. All authors contributed to the article and approved the submitted version.

## Conflict of Interest

The authors declare that the research was conducted in the absence of any commercial or financial relationships that could be construed as a potential conflict of interest.
